# Measurement of Blood Pressure Using an Arterial Pulsimeter Equipped with a Hall Device

**DOI:** 10.3390/s110201784

**Published:** 2011-01-31

**Authors:** Sang-Suk Lee, Dong-Hyun Nam, You-Sik Hong, Woo-Beom Lee, Il-Ho Son, Keun-Ho Kim, Jong-Gu Choi

**Affiliations:** 1 Department of Oriental Biomedical Engineering, Sangji University, Wonju 220-702, Korea; E-Mails: sonory@nate.com (I.-H.S.); wizkh@naver.com (K.-H.K.); 2 Department of Biofunctional Medicine and Diagnosis, Oriental Medical College, Sangji University, Wonju 220-702, Korea; E-Mail: omdnam@sangji.ac.kr; 3 Department of Computer Science Engineering, Sangji University, Wonju 220-702, Korea; E-Mails: yshong@sangji.ac.kr (Y.-S.H.); beomlee@sangji.ac.kr (W.-B.L.); 4 Department of Western-Eastern Biomedical Engineering, Sangji University, Wonju 220-702, Korea; E-Mail: choijong9@sangji.ac.kr

**Keywords:** Hall device, blood pressure (BP), arterial pulsimeter, pulse waveform

## Abstract

To measure precise blood pressure (BP) and pulse rate without using a cuff, we have developed an arterial pulsimeter consisting of a small, portable apparatus incorporating a Hall device. Regression analysis of the pulse wave measured during testing of the arterial pulsimeter was conducted using two equations of the BP algorithm. The estimated values of BP obtained by the cuffless arterial pulsimeter over 5 s were compared with values obtained using electronic or liquid mercury BP meters. The standard deviation between the estimated values and the measured values for systolic and diastolic BP were 8.3 and 4.9, respectively, which are close to the range of values of the BP International Standard. Detailed analysis of the pulse wave measured by the cuffless radial artery pulsimeter by detecting changes in the magnetic field can be used to develop a new diagnostic algorithm for BP, which can be applied to new medical apparatus such as the radial artery pulsimeter.

## 1. Introduction

Sensing-technology for measuring blood pressure (BP) and pulse can be used to provide a remote programmable service (monitoring exercise, diet, and medication) through an online network. Such a system provides services for management of disease or promoting health. Moreover, it is an important means of maintaining training momentum and managing the health of athletes. However, the currently available sensors are inconvenient to use because of restrictions on the position of the sensors and the time over which they can obtain readings [1,2]. In particular, it is impossible to measure BP and pulse without an unpleasant oppressive feeling due to the use of pressurization, and accuracy is remarkably low. If the accuracy of vital information is ambiguous, it cannot be used as baseline data to understand medical activity, so accurate measurements are a required precondition for the U-health industry [3,4].

Traditional technology used to measure pressure and pulse led to the concept of a measuring instrument incorporated into a portable device such as a watch or ring [1]. However, it is difficult to accurately measure the pulse using a traditional method when the subject is moving, because it is difficult to hold the sensor precisely in place and movement causes static even though the traditional pulse-taking method can measure BP and pulse if the pulse wave sensor is positioned over the aorta radialis. There are optical, electrocardiogram and pressure sensors in use as traditional pulse wave measuring sensors but the difficulty of obtaining measurements causes problems in manufacture and results in the problem of an oversized device. To solve these problems, it is necessary to develop a wrist-mounted measuring instrument for checking BP and pulse that has its sensor positioned on the perimeter of the radius at the wrist and can thus analyze BP or pulse at the aorta radialis using a Hall element.

The resulting data can be used as a basis for remote-controlled treatment and health care monitoring by sending the data through a wireless network based on the future U-health care network. The principal elements of pulse measurements by a human using the traditional pulse method are force and period of pulsatory motion, expansion of pulse, speed of contraction, palpation depth, width, and length of pulse [5,6]. The arterial pulsimeter developed by our group has the important technological ability to analyze and monitor BP and pulse by holding the sensor on the perimeter of the radius and the ulna at the wrist, and can thus effectively measure the pulse wave of the aorta radialis [7,8].

Under the traditional stable state, portable instruments developed to monitor BP and pulse rate are uncomfortable to use, and impractical for everyday life due to the pain caused by pressure on the skin. Recently, an arterial pulsimeter was developed and was on the market in Japan, but this still had some problems such as intermittent measurement of vital signals, pain when wearing it and its large size. Therefore, it was difficult for patients to wear this instrument continuously. Our model is portable in real life situations and is able to measure blood pressure and pulse at the same time without pain, using a non-pressurization method based on next-generation technology of the U-health industry. It is also possible to adjust the length of the strap and the pressure of the sensor so that the part in contact with the skin will press the radial artery lightly to classify to floating and sunken pulse. Therefore, the purpose of our research was to develop a method of reproducibly measuring BP and pulse rate better than a traditional method and thus to fill the dominant market position in this field [9,10].

To obtain accurate BP and pulse data, we have developed a pulsimeter that is a portable, simple, and miniaturized medical instrument based on a stable product. In addition, its circuit composition is designed to minimize power consumption and it can be worn on the wrist to obtain BP and pulse data continuously. The miniaturized pulsimeter is accompanied by software able to analyze multiple vital signs so as to enhance the commercial appeal of the pulsimeter. Using this software, medical treatment and prescriptions can be given over the network, and the software, which monitors regular exercise and diet, will send basic physical information concerning the individual’s health condition in real time. Therefore, our research led us to develop the first product with a magnetic Hall device sensor; this product can measure minute changes in the magnetic field using pulsatory motion and a permanent magnet. We also developed an algorithm to estimate BP by analyzing the pulse wave generated from data derived from patient tests.

## 2. Methods

### 2.1. Structure of a Radial Artery Pulsimeter with Hall Device

To develop a radial arterial pulsimeter, we analyzed what experimental data was required to determine the shape of the spatial pulse from a reading of the type of wave generated by a ranged Hall device with a constant arrangement.

The main material of the Hall device is a magnetic converted element composed of indium antimonide (InSb), indium arsenide (InAs), germanium (Ge), and silicon (Si) [11]. Because the size of the Hall electromotive force induced by a magnetic field can be used to determine magnetic force, the advantage is that the magnetic field of a micro portion of a magnetic field can be measured. In addition, when the alteration of the magnetic field results from changes in position, measuring their position is possible.

**Figure 1.**
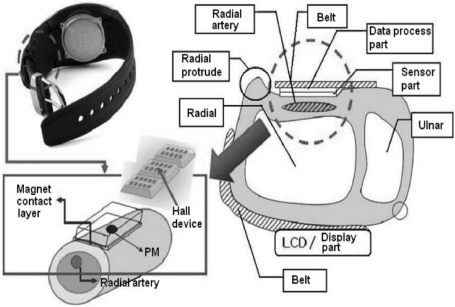
Diagram showing the basic structure of the wrist wearable radial arterial pulsimeter. Schematic cross-section of one form of the pulse-sensing and skin-contacting components of the arterial pulsimeter using multiple Hall devices and permanent magnets, respectively. The pressure chamber between the skin-contacting and pulse-sensing components is filled with air.

A schematic diagram showing the basic structure of the arterial pulsimeter is shown in Figure 1. The pulsimeter operates as follows: alteration of the voltage, caused by the Hall device, is interpreted as an electrical signal caused by a positional change of a permanent magnet situated on the radial artery. The electrical signal indicates the waveform of the pulse. Through the hardware of the circuit, the output signal is only the differentiated part of the signal resulting from the magnetic change. The signal process module shows the shape that represents a picture of the pulse according to the change of position of the magnetic field analyzed by the software.

**Figure 2.**
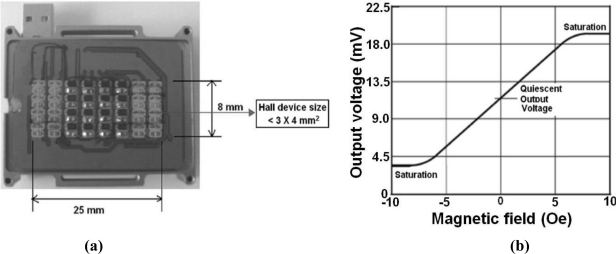
**(a)** PCB pulse-sensing component mounted with a multiple 5 × 9 = 45 array of Hall devices corresponding to the permanent magnets underneath. **(b)** A graph of output voltage versus magnetic field for the commercial, A3515-and A3516-type Hall device showing high sensitivity of 1.2 mV/Oe and linearity in the magnetic field and temperature range from −5 Oe to +5 Oe and from −40 °C, to +150 °C, respectively.

The size of the multiplex Hall device and its sensitivity to a magnetic field enables the pulsimeter to measure a pulse wave at a radial artery position based on its semiconductor, which is the real shape of a printed circuit board (PCB) with semiconductor multiplex Hall device and the property of a curve, which is 3 × 4 mm^2^ and 1.2 mV/Oe, as shown in Figure 2.

### 2.2. Experimental Procedure and Data Acquisition

The imaging circuit diagram of system by computer, read 36 Hall devices of three sets according to the fact that magnetic Hall device is a set. Generally, through hardware, the signal from the Hall device is delivered to the software by converting it into a waveform and then combining all the waveforms of peak value and converting them into a video signal that constitutes the monitoring structure. Each component of the hardware functions as follows. The electrical signal entering through three multiplexes in the Hall device array creates a minute parallel condition through the Wheatstone bridge circuit. This data is also saved to a memory device through an amplifier circuit and a bandwidth filter circuit. The data of 30 frames per second (FPS) is rapidly processed. Output signals of the processing hardware are delivered to the circuit part of the software, which is connected to RS232C, and the signal is converted to the MatLab program through the matrix so that an image is displayed on the monitor.

**Figure 3.**
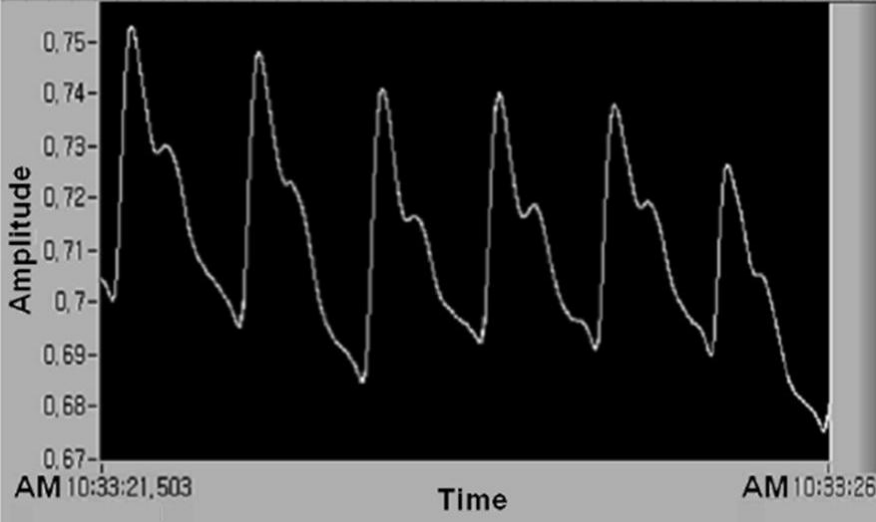
Typical pulse waveform of 1 point obtained by the compositional analysis of pulse signal of the clinical product testing of the pulsimeter using Hall device. Example of measuring time (seconds) versus temporally typical signal of 1 point pulse obtained from the analysis of an arbitrary pulse signal of 1 position of a small permanent magnet.

The pulse wave is amplified through measurement of the number of amplifications, and the pulse wave signal is received through automatic zero point setting, large output, noise filter, and attenuation. The waveform is received again with a lower power of computer simulation through the output of RS232C and 30 FPS by 12-bit resolution. To analyze the waveform of the pulsimeter used in the multiplex Hall device, we analyzed the saved data of waveform analysis at the number 32 channel, which is used in LabVIEW, linked real-time image, two dimensions of multiplex Hall device, sequential unlimited storage data, and Matlab. Figure 3 shows that typical pulse wave (number of pulses per minute = approximately 64) obtained by five dots moving through an average filter. The waveform shown in Figure 3 was obtained using a Hall device of sensing a minute magnetic field change by a permanent magnet. Thus, we measured the pulse wave in non-pressurization method and found diagnostic algorism of estimated BP based on the analyzed data.

## 3. Results

### 3.1. Testing of the Radial Artery Pulsimeter

The autonomic nervous system adjusts the BP in response to signals from the baroreceptors in the walls of the large systemic arteries and the chemoreceptors in the carotid and aortic body. The chemoreceptors respond smoothly and weakly to oxygen lack, carbon dioxide excess or hydrogen ion excess while the baroreceptors respond rapidly and strongly to direct BP changes which induced mainly by the postural change. The heart rate also increases to increase the supply of blood. Thus, pulse frequency also increases so it further increases the BP (the case that restrictive use of patient with arrhythmia and angina). Division of the pulse waveform brings the time difference of the reflected wave, which reflects to the lower body from the main pulse wave of the moving radial artery from the heart. Thus, the individual features of blood vessels and blood pressure can be evaluated.

**Figure 4.**
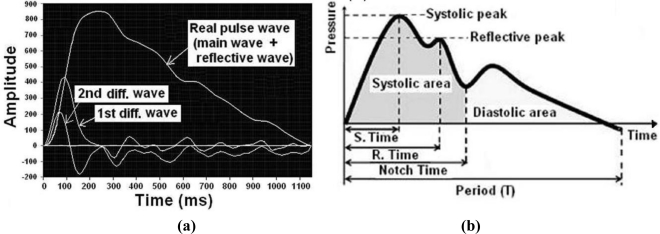
**(a)** Typical real features of a pulse wave and a first pulse wave combined to provide the main pulse and the reflective pulse. **(b)** Regression analysis equation for the estimated algorithm of blood pressure needs the following 6 major correlative factors; (1) the period of the pulse wave, (2) the time of the systolic period, (3) the reflective time, (4) the notch time, (5) the ratio of area (systolic period area/diastolic period area), and (6) the increasing pressure index (reflective wave peak/systolic wave peak).

Figure 4 is an example of application of the algorithm to estimate blood pressure and it shows the relationship of the main correlation factor about the regression analysis equation for the estimated algorithm of BP. We show that the necessary main correlation factors for deducing the regression analysis equation for the estimated algorithm of blood pressure are the time of the systolic period, reflective time, notch time, acceleration of the pulse wave, ratio of area (systolic period area/diastolic period area), and increasing pressure index (reflective wave peak/systolic wave peak).

**Figure 5.**
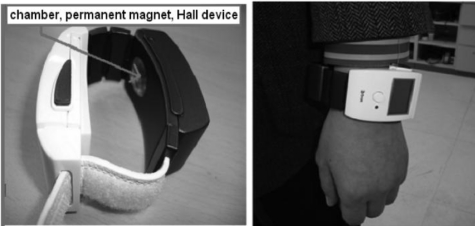
Photographs of the arterial pulsimeter with Hall device in clinical tests. Here the weight and power consumption of the wrist wearable, radial arterial pulsimeter are 80 g and 1.65 W, respectively. The product testing of the proposed arterial pulsimeter and the measuring feature, which are a wristwatch or bracelet, transfer the increased pressure to the skin-contacting part intact when the pressure of the constant pressure chamber is increased.

The first radial artery pulsimeter we have developed is shown in Figure 5 and uses a Hall device to obtain the data. A permanent magnet and a Hall device placed inside an air bladder are arranged to safely contact the biggest pulse spot on the radial artery. The pulsimeter weight has been reduced to about 80 g, including the battery. Our tests showed that the amount of electrical power provided by a lithium battery is less than 1.65 W (Output current 500 mA, 3.4 V) after wearing the pulsimeter.

This device is able to save the values of high/low BP measurements for 1 h, if the battery is 100% recharged. Increases in the heart rate calculated from the estimated BP algorithm are based on the fact that blood supply and pulse rate are causes of the increased heart rate. We were able to distinguish a normal, healthy person from a high BP patient with relation to the pulse waveform containing systolic and diastolic BP; this enabled us to distinguish the groups on the basis of the shape of the pulse wave.

### 3.2. Major Correlated Parameters of the Estimated Blood Pressure through Analysis of the Pulse Wave

We chose the representative pulse wave automatically or designated a specific point to use the saved pulse data using the technology of the estimated BP algorithm. The data saved automatically was used to extract eight factor values through an automatic calculation. If an automatic input does not work, the automatic calculation can be obtained by manual input; the automatic calculation then decides a representative pulse wave and processes it to get its frequency and pulse rate, then specifies the end and start point of the frequency of the pulse wave located right before the fifth and sixth zero point of the first differential wave. The diastole is justified as the start point of the representative pulse wave and the systolic period is appointed as the first zero point of the first differential wave. The systolic peak (or augmentation point) is appointed as the sixth differentiation’s second or third zero point of normal BP’s pulse wave and the notch point is appointed as the zero point of the third or fourth of sixth differentiation and pulse waves of high BP [6].

The participants in the clinical trial to establish the algorithm for blood pressure estimation consisted of thirteen adults of varying ages, twenty to sixty, who were registered students, graduate students or faculty members of Sangji Oriental Medical Industry Department (seven males, six females). The experimental method was to measure the pulse wave with a non-pressurization method pulsimeter over about 5 s to get an output value of the pulse wave. Immediately after obtaining the pulse wave, BP was measured directly using an electrical sphygmomanometer on the right arm and a mercury one on the left arm. The output data were used to choose 3 pulse waves.

Pulse wave factors and analysis of the pulse data to generate a regression equation for estimating blood pressure involves eight pulse-related factors; (1) the index of systolic (augmentation) pressure, (2) the index of notch point (incisura point), (3) the time of arrival of the reflected wave, (4) the pulse rate, (5) the ratio of the value between the first differentiation peak, (6) the ratio of the value between the second differentiation peak and the valley of the pulse wave, (7) the time ratio of systolic area and frequency, and (8) the extent ratio of diastolic area and systolic area. The value of the estimation point and automatic detection, which come from the Y-value between the first differential wave and the zero point of the pulse wave, and the differential wave of the representative pulse wave compensated each other. To analyze these data, we used Statistical Package for the Social Sciences (SPSS) software.

## 4. Discussion

The main characteristic of the pulse wave used to calculate the estimated BP is extracted by applying the difference between the ratio of the arrival time of the progressive wave and that of the reflected wave and this must be performed each time, because the air inside the air bladder containing the permanent magnet can be changed by relative pressure. To obtain a more precise value of estimated BP, we divided data from the clinical trial into five modes separated by 10 units between 20 s and 60 s. Through analysis of these data, the regression of systolic and diastolic estimation of blood pressure, which considers correlation factors, is represented by Equations (1) and (2) below. Here, nine important parameters are defined as follows: (S_BP_), systolic blood pressure; (D_BP_), diastolic blood pressure; (S_T_), time of systole; (N_T_), time of notch point; (a), peak value of pulse wave acceleration; (b), minimum value of pulse wave acceleration; (R_T_), time of reflected wave; (P_N_), pulse rate; and (N_1_), index of Notch point. In these equations, the unit of blood pressure is mmHg:

(1) $$S_{\mathrm{BP}}=-233.072\times S_{T}+220.77\times N_{T}+21.22\times \left(\frac{b}{a}\right)-297.958\times R_{T}+0.096\times P_{N}+171.19$$

(2) $$D_{\mathrm{BP}}=12.65\times N_{1}+5.866\times \left(\frac{b}{a}\right)+61.23$$

We compared the measured value and the estimated value of blood pressure obtained from clinical trial data of 41 people. Table 1 shows the comparison between the measured value and the estimated value of BP from thirteen clinical patients. The standard deviation of the error in the estimated peak (systolic) BP and the minimum (diastolic) BP obtained from regressions (1) and (2) are calculated as 8.3 and 4.9. The error in peak blood pressure is a little higher than the International Standard margin of error for BP (±10), but the error in the minimum BP value is lower than the International Standard for BP [12]. If we are able to build up more data and thus develop an algorithm that is able to decrease the error of systolic BP readings, our pulsimeter will be suitable for commercial applications. Therefore, we could obtain valid values when we classified a pattern that applies the algorithm to earn accurate conclusion by age, and we are able to construct the regression by categorizing it from pulse waves with a similar pattern.

If patients always wear the wrist wearable pulsimeter, the frequency of diagnosis will rapidly increase and the rate of early detection of diseases will be higher. Furthermore, patients may benefit from new information about caring for their health and health problems at home. In addition, we will be able to graft BT onto IT to develop a new U-healthcare system that can save continuous vital sign data and analyze that data remotely through a wireless network, helping athletes to keep in shape and providing an efficient medical service to the chronically ill.

According to the U-health industry, the new medical paradigm, such a medical service will provide diagnosis, monitoring, and advice, not only in hospitals, but also in everyday life. Domestic companies are urged to develop this technology, because foreign global companies such as Intel, IBM and others are already entering this field and expanding in this area. The U-health industry will be very important in reducing medical expenses in our society as a next generation growth industry, because this industry is important for an aging society such as ours, which has rapidly increasing medical expenses. As the market for the U-health industry is expected to grow continuously, the main technologies, and the accuracy and convenience of sensing the medical information necessary to care for an individual’s health, must be standardized for developing products. In the bigger picture of the U-health industry, developing a sensing technology that monitors an individual’s health and measures BP and pulse of a patient is necessary to enlarge the range of medical services. This makes it possible that the chronically ill, the old and the infirm can measure and monitor their BP or pulse repeatedly, enabling management of an optimized service that prevents disease.

## 4. Conclusions

We have invented a miniaturized portable pulsimeter that can be used to obtain accurate BP and pulse measurements. In addition, the circuit composition was designed to minimize electricity consumption and was able to obtain continuous measurements of BP and pulsation data using a convenient wrist wearable non-pressurization method incorporating a pulse wave analysis algorithm. The pulse wave data obtained from a clinical test of thirteen subjects resulted in the regression analysis equation of the estimated algorithm with the pulse wave being the determinant of the correlation factor. We compared the values of estimated blood pressure from pulse wave data collected by the non-pressurization pulsimeter at the left wrist for 5 s with the measured values of BP obtained by an electron sphygmomanometer at the right upper arm and a mercury sphygmomanometer at the left upper arm; we found that the standard deviation of the minimum and maximum BP obtained by the non-pressurization pulsimeter was 8.3 and 4.9, respectively. Since the value of the standard deviation was close to the limit on the International BP Standard, this device could be commercialized as a non-pressurization BP and arterial pulsimeter. Therefore, through the analysis of non-pressurization pulse wave using a magnetic Hall device that perceived a minute magnetic field change of permanent magnet, we proposed the new estimated BP diagnostic algorism of Traditional Chinese Medicine.

## Figures and Tables

**Table 1.** Comparison of the measured value and the estimated value of BP applied to the clinical data.

**BP Classification**	**Systolic Blood Pressure (SBP)**			**Diastolic Blood Pressure (DBP)**		

**Clinical Number**	**Estimated Value**	**Measured Value**	**Error**	**Estimated Value**	**Measured Value**	**Error**
1	133.0	145.0	12.0	64.0	63.0	−1.0
2	131.6	140.0	8.4	65.0	63.0	−2.0
3	135.1	142.0	6.9	64.3	71.0	6.7
4	135.4	140.0	4.6	63.8	67.0	3.2
5	132.9	142.0	9.1	63.6	68.0	4.4
6	132.8	135.0	2.2	63.5	71.0	7.5
7	135.0	137.0	2.0	62.6	63.0	0.4
8	133.6	135.0	1.4	65.1	67.0	1.9
9	133.3	136.0	2.7	62.1	64.0	1.9
10	132.9	133.0	0.1	60.5	58.0	−2.5
11	128.3	130.0	1.7	63.0	61.0	−2.0
12	133.1	135.0	1.9	62.3	54.0	−8.3
13	129.6	105.0	−24.6	59.7	50.0	−9.7
**Standard Deviation**			**8.3**			**4.9**
